# The formation of acetylcholine receptor clusters visualized with quantum dots

**DOI:** 10.1186/1471-2202-10-80

**Published:** 2009-07-16

**Authors:** Lin Geng, Hailong L Zhang, H Benjamin Peng

**Affiliations:** 1 Department of Biology, Hong Kong University of Science and Technology, Clear Water Bay, Kowloon, Hong Kong

## Abstract

**Background:**

Motor innervation of skeletal muscle leads to the assembly of acetylcholine receptor (AChR) clusters in the postsynaptic membrane at the vertebrate neuromuscular junction (NMJ). Synaptic AChR aggregation, according to the diffusion-mediated trapping hypothesis, involves the establishment of a postsynaptic scaffold that "traps" freely diffusing receptors into forming high-density clusters. Although this hypothesis is widely cited to explain the formation of postsynaptic AChR clusters, direct evidence at molecular level is lacking.

**Results:**

Using quantum dots (QDs) and live cell imaging, we provide new measurements supporting the diffusion-trap hypothesis as applied to AChR cluster formation. Consistent with published works, experiments on cultured Xenopus myotomal muscle cells revealed that AChRs at clusters that formed spontaneously (pre-patterned clusters, also called hot spots) and at those induced by nerve-innervation or by growth factor-coated latex beads were very stable whereas diffuse receptors outside these regions were mobile. Moreover, despite the restriction of AChR movement at sites of synaptogenic stimulation, individual receptors away from these domains continued to exhibit free diffusion, indicating that AChR clustering at NMJ does not involve an active attraction of receptors but is passive and diffusion-driven.

**Conclusion:**

Single-molecular tracking using QDs has provided direct evidence that the clustering of AChRs in muscle cells in response to synaptogenic stimuli is achieved by two distinct cellular processes: the Brownian motion of receptors in the membrane and their trapping and immobilization at the synaptic specialization. This study also provides a clearer picture of the "trap" that it is not a uniformly sticky area but consists of discrete foci at which AChRs are immobilized.

## Background

The formation of high-density clusters of transmembrane neurotransmitter receptors is a key event in the differentiation of the postsynaptic membrane of chemical synapses. At the neuromuscular junction (NMJ), nicotinic acetylcholine receptors (AChRs) are clustered to near crystalline density of 10,000/μm^2 ^[1,2]. The mechanism underlying the assembly of this elaborate membrane specialization has been extensively studied during the past three decades, and results from cellular, biochemical and molecular genetic studies have established the following paradigm with regards to synaptic AChR aggregation [2-4]: During early stages of synaptogenesis, growth cones of motor axons approach skeletal muscle fibers and locally deposit the heparan sulfate proteoglycan agrin at contact sites. Agrin activates the muscle-specific receptor tyrosine kinase MuSK to initiate a signaling cascade that leads to AChR clustering.

Over the past decade a significant amount of knowledge has accumulated regarding the molecular nature of agrin-MuSK signaling, but relatively little is known about how AChRs are actually aggregated into a cluster. Thirty years ago, Edwards and Frisch [5,6] proposed the diffusion-mediated trapping model to explain the local concentration of AChRs at the NMJ. According to this model, receptors inserted into the membrane are free to diffuse until they move into a "sticky zone" in the subsynaptic region opposite to the nerve terminal and become confined there. The diffusion-trap model, which has been the underlying hypothesis for understanding the final steps in the formation of AChR clusters, is supported by several lines of experimental data. For example, studies using the fluorescence-recovery-after-photobleaching (FRAP) technique have demonstrated the diffusible nature of AChRs in cultured muscle cells and their immobility within clusters [7-9]. Moreover, synaptogenic signaling has been shown to cause the assembly of an F-actin cytoskeleton necessary for the formation of AChR clusters [10-12]. This cytoskeletal specialization presumably interacts through linker proteins with AChRs and their associated cytosolic protein rapsyn to mediate receptor trapping. A direct test of this hypothesis at the single-molecular level, however, is still lacking.

Recent advances in quantum dot (QD) technology have made it possible to track the movement of single molecules. QDs are fluorescent semiconductor nanocrystals that are considerably brighter and more photo-stable than conventional fluorophores [13]. When specific proteins are coupled to their surface, QDs become excellent probes for molecular localization in cells, as demonstrated by their recent use in tracking glycine receptors [14,15] and ion channels [16,17]. at neuronal synapses. In this study, we used QDs to follow the movement of diffuse AChRs on the surface of cultured muscle cells and to examine their clustering in response to synaptogenic stimuli. This allowed us to visualize the diffusion of AChRs in the plane of the muscle membrane and to directly test the diffusion-trap hypothesis. Here we present our analyses of the behavior of single AChRs and their trapping in muscle cells during cluster assembly.

## Methods

### *Xenopus *cell cultures and induction of AChR clustering

Myotomal muscle cells and spinal neurons were isolated from stage 20–22 *Xenopus laevis *embryos according to previously published methods [18]. Cells were plated on glass coverslips in Steinberg's solution, consisting of 60 mM NaCl, 0.67 mM KCl, 0.34 mM Ca(NO_3_)_2_, 0.83 mM MgSO_4 _and 10 mM Hepes at pH 7.4, supplemented with 10% L-15 (Leibovitz) medium, 1% fetal bovine serum, 100 U/ml penicillin. The cultures were maintained at 23°C.

Polystyrene latex beads (10 μm diameter; Polysciences, Warrington, PA) were coated with recombinant heparan-binding growth associated molecule (HB-GAM) (kindly provided by Dr. Heikki Rauvala, University of Helsinki) and applied to muscle cells as described [19]. AChR clustering induced by the beads was examined within a few hours of bead addition. To prepare nerve-muscle cocultures, spinal neurons dissociated from neural tubes were seeded onto 3-day old muscle cells; nerve-muscle contacts were examined one day later.

### Live imaging of AChR clustering

For AChR labeling, biotin-conjugated α-bungarotoxin (biotin-BTX) and Alexa 488-conjugated α-bungarotoxin (Alexa 488-BTX) (Invitrogen) were diluted into culture medium at concentrations of 0.5 nM and 25 nM respectively. Streptavidin-conjugated QD655 (Invitrogen) was diluted into culture medium at the final concentration of 2.5 nM. Cells were incubated with biotin-BTX and Alexa488-BTX (excitation wavelength 488 nm) for 5 min, washed three times over 30 min with culture medium, incubated with QD655 (maximum emission 655 nm) for 10 min, and then washed again extensively with culture medium before observation using a custom-built chamber. The 1:50 ratio of biotin-BTX and Alexa 488-BTX in the first labeling step resulted in very low density of AChRs tagged by the biotin-BTX-streptavidin QD complex. The labeling was carried at room temperature.

For labeling GM1 ganglioside, a process similar to that for AChR was used, except that 50 ng/ml biotin-conjugated cholera toxin B subunit (biotin-CTX) (Sigma-Aldrich) reconstituted in 5% BSA was applied instead of biotin-BTX.

Cells were imaged at room temperature with an Olympus IX70 inverted microscope equipped with 60× (N.A. 1.40) objective. QD655 and Alexa488-BTX were observed with fluorescence filters for rhodamine and FITC respectively. The filter combination for QD imaging was as follows: excitation filter with band-pass wavelength of 510–550 nm, emission filter with long-pass wavelength of 590 nm. Images were captured with a 500 ms exposure using a cooled digital CCD camera (Hamamatsu ORCA II-ER, C4742–98) interfaced with an electronic shutter (Sutter Instrument) under the control of Metamorph software (Universal Imaging). QDs were also tracked with 100 ms exposure time, the lower limit of our imaging system. The diffusion coefficients calculated from image stacks obtained with 500 ms and 100 ms exposure time were similar.

### Receptor tracking and data analysis

Single-molecular tracking was performed using the "track point" function of Metamorph software. This feature allows single points within each image frame of a time-lapse series to be tracked and enables the measurements of paths, positions and velocities of these points. Our labeling strategy was aimed at achieving low QD density to avoid receptor crosslinking. Multiple QDs within a time-lapse series were individually analyzed by repeating the track-point function to ensure tracking accuracy. Data were then incorporated into a spreadsheet for further analysis. Because of the intrinsic blinking property of QDs, single QDs sometimes became invisible for short periods of time and reappeared later during time-lapse recordings. For short-term experiments (50 frames, 5–25 sec), only data from non-blinking periods were used.

Mean square displacement (MSD) and diffusion coefficients were calculated according to established formula [10,20]:



where (*x*_*n*+*i*_, *y*_*n*+*i*_) is the position of the QD following a time interval of *nt *(*t *is the time interval between successive measurements) after starting at position (*x*_*i*_*, y*_*i*_). *N *is the total number of positions recorded; *n *ranges from 1 to *N-1*. QD position data at each time point were put into a spreadsheet generated by Metamorph software and then fed into software written in-house which calculated *MSD *automatically from data in the spreadsheet according to above formula. The statistical significance of differences was quantified with Student *t*-test using SigmaPlot software (SPSS, Chicago, IL).

Diffusion coefficients were calculated according to the formula:



where *D *is the diffusion coefficient and *t *is the elapsed time interval.

For QD tracking lasting more than 10 min, it was not possible to circumvent the blinking problem. To analyze tracks in which QD points were missing in three frames or less, an interpolation algorithm was developed to estimate the missing positions. First, *MSD*_*b *_from points up to the blinking was calculated. Equations were then set up to calculate the x and y positions of the invisible points that minimized the difference between *SD *(square displacement between the invisible and visible points before and after blinking) and *MSD*_*b*_. To assess the validity of this algorithm, a simulation based on a 100-point track was conducted. We randomly deleted one, two or three points from this track to simulate QD blinking and the missing points were then filled in by the algorithm. The diffusion coefficients calculated from the filled-in track showed no significant difference from that calculated from the original track.

## Results

### Visualization of AChRs with QDs

**Figure 1:**
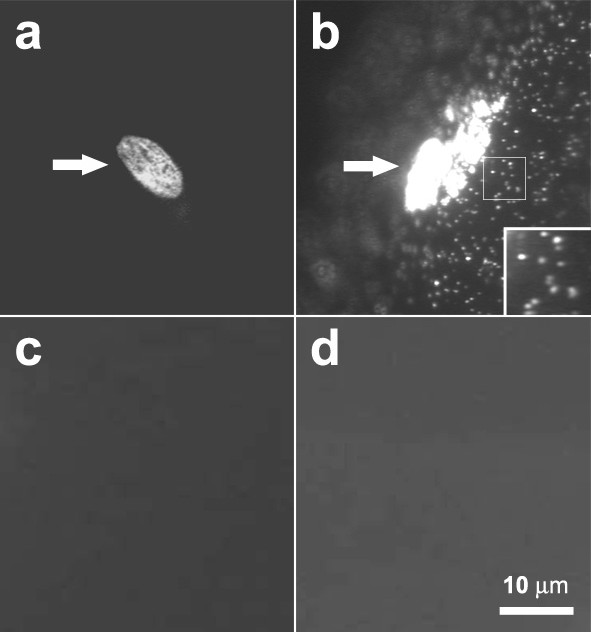
**QD labeling of AChRs**. (a) Alexa488-BTX and (b) BBQ. Pre-patterned AChR clusters (arrows) were seen with both labels, but diffuse AChRs were only seen after BBQ labeling as individual fluorescent dots. (c) Pretreatment with 5 μM unlabeled BTX for 30 min before BBQ labeling. (d) QD labeling without biotin-BTX. Scale bar, 10 μm.

In cultured muscle cells AChRs often spontaneously form clusters in the absence of synaptogenic stimulation. These pre-patterned clusters (also called hot spots) can be readily visualized by labeling cultures with fluorescent α-bungarotoxin (BTX), as shown by the example in Fig. 1a where Alexa488-BTX was used to label muscle cells. With this method, however, individual non-clustered AChRs distributed diffusely on the cell surface cannot be detected. To visualize the diffuse receptors, muscle cells were labeled with biotinylated BTX followed by streptavidin-conjugated QDs, or BBQs for biotin-BTX/QDs. BBQs strongly labeled pre-patterned AChR clusters as well as non-clustered AChRs on the muscle surface (Fig. 1b). Thus, QDs enabled both aggregated and non-aggregated AChRs to be probed.

BBQ-labeling specificity was confirmed in several ways. No QD signal was present when BBQs were applied after muscle cells were pretreated with 5 μM unlabeled BTX for 30 min (Fig. 1c) or when cells were treated with QDs but without biotin-BTX (Fig. 1d), and non-muscle cells found in our cultures were not labeled by BBQs (data not shown). These results demonstrated that BBQs selectively marked muscle AChRs. We also noted that pre-patterned AChR clusters on the top surface of muscle cells were strongly labeled by QDs but clusters on the bottom facing the coverglass substratum were not, presumably because QDs were excluded by the tight cell-substratum attachment at these sites (a gap of 10–15 nm; [21,22]). Although diffuse AChRs on cell bottom could be labeled by BBQs, in this study we focused on tracking only AChRs present on the top surface of muscle cells.

### Tracking the movement of AChRs

When a saturating concentration of BBQ was used to label all diffuse AChRs, it was not possible to track individual receptors unambiguously due to their high density. A range of BBQ concentrations was thus tested to obtain the optimal labeling condition. A mixture of Alexa488-BTX and biotin-BTX at a ratio of 50:1 yielded low BBQ density at the cell surface and enabled accurate tracking of single molecules.

The high fluorescence stability of QDs allowed their observation over long periods. Under our experimental conditions cells were healthy during time-lapse recordings for more than 30 min as judged by the intactness of the characteristic cross-striations in phase optics and by diffusion coefficient measurements (described below). Furthermore, for analyses we only used data obtained during the first 10–20 min recording from cells that remained healthy at the end of the observation period. We also used muscle cells maintained in cultures for different periods of time and found that under identical BBQ labeling conditions, surface receptor density was independent of culture age up to three weeks, but the fraction of immobile AChRs increased with time (see below). Results below were all obtained using cell cultures less than one week old.

**Figure 2:**
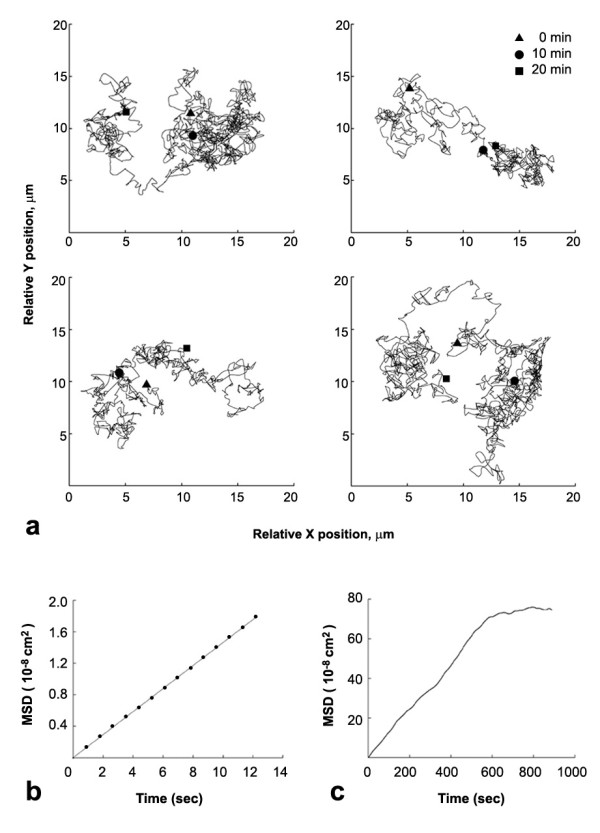
**Tracking the movement of single AChRs with QDs**. (a) Sample trajectories of single BBQs during a 20 min interval with positions at 0, 10 and 20 min marked. (b, c) Sample MSD plot of a single BBQ during short or long time interval.

To examine the movement of QD-labeled AChRs, time-lapse recording was carried out. Taking advantage of the fact that only single QDs blink, we were able to identify AChRs linked to single BBQs (see Discussion for further details) and track their trajectories on the muscle surface (Fig. 2a). BBQ-labeled receptors underwent random movement on the cell surface, and to quantify this movement mean square displacement (MSD) was calculated. As shown in Fig. (2b and 2c), the early phase of the MSD plot lasting up to 10 min was typically linear, which is characteristic of particles undergoing free Brownian motion. Over 20 min BBQs covered an area as much as 20 × 20 μm^2 ^but the net distance traversed (between the beginning and end point) was typically much less (Fig. 2a).

**Figure 3:**
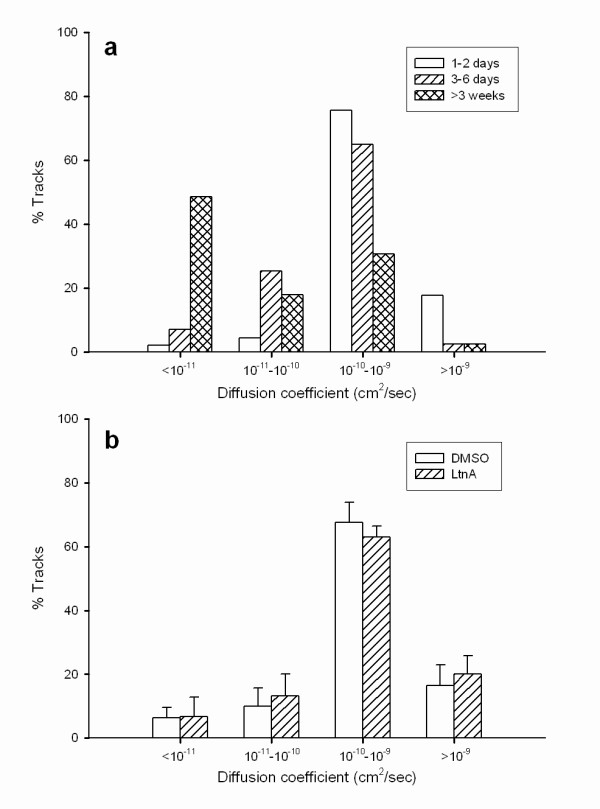
**The distribution of AChR diffusion coefficients in cultured Xenopus muscle cells**: (a) changes during development, (b) LtnA (40 μM) treatment.

From the linear portion of the MSD plot, the diffusion coefficient (*D*) of single BBQs was calculated to range from 10^-12 ^to 10^-9 ^cm^2^/s. In muscle cultures up to 6 days old, the majority of receptors moved with *D *on the order of 10^-10 ^cm^2^/s, while a small percentage of receptors showed faster movement (> 10^-9 ^cm^2^/s) (Fig. 3a). The proportion of fast moving receptors was highest in cultures less than 2 days old but decreased subsequently with an increase in the population of slower moving receptors, with *D *on the order of 10^-11 ^cm^2^/s (Fig. 3a). We also found a population of immobile or nearly immobile receptors with *D *less than 10^-11 ^cm^2^/s (Fig. 3a) which was rarely detected in cultures less than 2 days old but increased with culture age to reach 50% of total after 3 weeks. This suggests that diffuse AChRs become increasingly restrained in their membrane environment with development. In this study, data were collected mainly from cultures 3–6 days old.

Although the *D *values of single AChRs varied from cell to cell, they were remarkably constant when measured from the same cell (data not shown), and for a single AChR, *D *values calculated from different parts of its trajectory were also essentially the same. For example, *D *values calculated for a single AChR from one 17-min trajectory were 9.9 × 10^-10^, 8.5 × 10^-10 ^and 1.2 × 10^-9 ^cm^2^/sec during the early (< 30 sec), intermediate (8–8.5 min) and late (16.5–17 min) stages respectively. During the time-lapse recording, some cells became non-viable as shown by obvious morphological changes such as sudden shrinkage or loss of cross-striation, presumably due to photo-damage. This transition was always associated with a sudden cessation of BBQ movement.

### AChR movement at pre-patterned clusters

**Figure 4:**
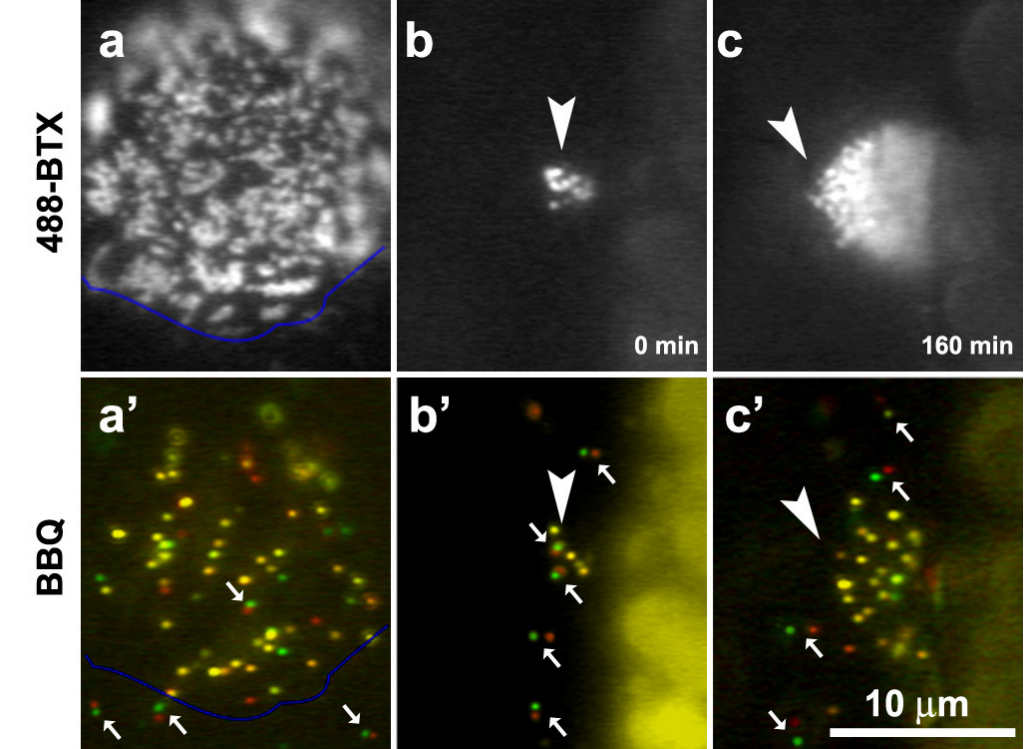
**AChR movement within or near pre-patterned clusters**. Consecutive time-lapse frames (separated by 2.5s) were pseudo-colored in red and green and superimposed. Yellow dots: immobile BBQs; paired green/red dots (arrows): mobile BBQs; un-paired green or red dots: BBQs that blinked during the recording and thus only one color was recorded. (a, a') A well-developed cluster on a 3-day old muscle cell shown by Alexa488-BTX (a) and BBQ (a') labeling with the blue lines outlining the lower edge of this hot spot. While most of the receptors within the cluster were immobile (yellow dots), those outside were mobile (small arrows). (b, b' and c, c') Development of an AChR cluster (arrowhead) in a 1-day old culture during a 3 hr recording period. b, c: Alexa488-BTX; b', c': BBQ.

To study the behavior of single AChRs during the formation of clusters, we examined BBQ movement at or near these sites. First, pre-patterned AChR clusters in non-innervated muscle cells were studied. These prominent and structurally complex clusters were observed in nearly all cells after three days in culture and within them most BBQs were stationary or nearly immobile (with *D *of 10^-12 ^cm^2^/s). Fig. 4 shows BBQ movement (a'-c') within or near clusters identified by Alexa488-BTX (a-c). The BBQ images (Fig. 4a'–c') were obtained by superimposing two frames of a time-lapse recording separated by 2.5 s, with the first image pseudo-colored in green and the second in red. Immobile receptors appear as yellow dots and mobile ones as green or red dots. Most AChRs within the cluster domain were immobile (Fig. 4a, a') but those outside were mobile (arrows at the bottom of Fig. 4a') with *D *on the order of 10^-10 ^cm^2^/s. Movement of individual BBQs into and out of a cluster was infrequently observed.

To visualize the behavior of single AChRs at developing pre-patterned clusters, we studied muscle cultures 1–2 days old when the clusters present were much smaller than mature clusters (Fig. 4b). The clusters grew at a rate of 0.2 ± 0.07 μm^2^/min over 2–3 h to reach a size of 5–6 μm in diameter. Single BBQs remained mobile within and around the clusters before they reached the final size (arrows in Fig. 4b, b'), but became mostly immobile within but not outside cluster confines at the end of this process (arrows in Fig. 4c, c', same cluster as in 4b, b' at a later stage). These observations suggest that a highly localized mechanism for immobilizing diffuse AChRs is set up at the onset of cluster formation and it grows through an expansion of the membrane domain for AChR entrapment.

### A test of the diffusion-trap hypothesis: AChR clustering induced by beads

Although pre-patterned AChR clusters are reliably found in cultured muscle cells, their position cannot be predicted. Therefore, to further understand how single AChRs are recruited into clusters, we studied clusters induced by beads coated with heparin-binding growth-associated molecule (HB-GAM). HB-GAM-coated beads focally induce AChR clusters with high fidelity, and with their use both the onset and location of clustering can be precisely marked [19]. By fluorescent BTX labeling, clusters become detectable within two hours at bead-muscle contacts, and the density of AChRs (as reflected by fluorescence intensity) at these clusters increases until reaching saturation after overnight bead stimulation.

**Figure 5:**
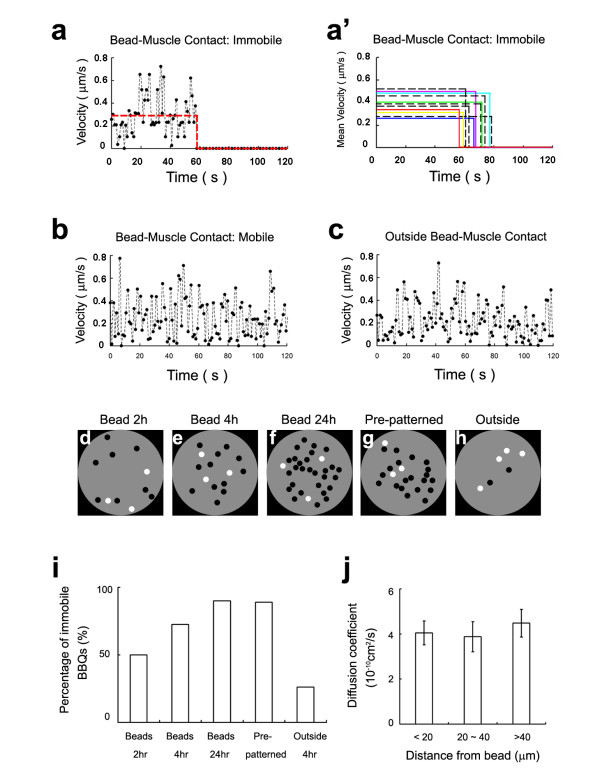
**AChR movement during the formation of bead-induced clusters**. (a-c) Instantaneous velocity plots of single BBQs at or away from a bead-induced AChR cluster. (a) Movement of a receptor at bead-muscle contact and its trapping at the 60 sec. The red line shows the mean velocity before and after trapping. (a') Additional examples of BBQ trapping. Only mean velocities of individual BBQs are shown. Color solid lines: BBQs underneath beads; black dashed lines: BBQs at the rim of beads. (b) Example of a freely moving BBQ at bead-muscle contact. (c) A freely moving receptor outside bead contact. (d-f) Schematic representation of immobile (black dots) and mobile (white dots) BBQs at a single bead-induced AChR cluster at 2, 4 and 24 hr after bead addition, showing increasing number of immobile receptors. (g) Immobility of BBQs at a pre-patterned cluster, in contrast to mobile ones outside the cluster area (h). (i) Quantification of receptor mobility based on diffusion coefficient measurements. Receptors with *D *less than 10^-11 ^cm^2^/s are designated immobile and those with *D *above this value are considered mobile. BBQs outside bead-muscle contact were also tracked 4 hr after bead addition ("outside 4 hr"). (j) Diffusion coefficients of mobile BBQs at various distance away from bead-muscle contacts (mean ± sem; number of tracks analyzed: 19, 11 and 21 from near to far).

AChR movement at developing bead-induced clusters was followed using BBQs and time-lapse recording. Single BBQs within these clusters were individually tracked for 2 min or longer at three different time points (2, 4 and 24 hr) after the establishment of bead-muscle contacts. An example of the movement history of one such single BBQ in a bead-contact area (Fig. 5a) shows that the BBQ displayed continuous random diffusion with variable instantaneous velocity for 1 min but then suddenly stopped at the 1 min mark and remained immobile afterwards; the BBQ's mean velocity while in motion is indicated by the horizontal line. The colored lines in Fig. 5a' show other examples of BBQ movement and trapping at bead-muscle contacts, with each line indicating the mean velocity of one BBQ.

To eliminate the possibility that BBQs were immobilized at bead-induced clusters as a consequence of the tight cleft space between the bead and the cell surface, QD movement was also recorded at bead edges. Although this zone is at the periphery of bead-muscle contacts, AChRs are clustered there [23], and BBQs at these sites (Fig. 5a'; black dashed lines) moved and then suddenly stopped and remained stationary afterwards during the recording period. Thus, immobilization of BBQs is not due to simple mechanical restraint within the bead-cell cleft space.

A second subset of BBQs remained mobile within the bead-muscle contact region throughout the recording period (as shown by the velocity plot in Fig. 5b) and sometimes exited this area with time. Although these mobile AChRs were inside the area of cluster formation, they were indistinguishable in their continuous movement from receptors that were outside the bead-muscle contact (Fig. 5c). However, the fraction of immobile AChRs (*D *< 10^-11 ^cm^2^/s) increased over time within the bead-induced clustering domain. Sample recordings are graphically shown in Fig. 5d–h and quantified in Fig. 5i. In consistency with our previous finding that AChR clustering starts at bead-muscle contacts within minutes, we observed that the fraction of immobile AChR rose significantly (to 50%) during the first two hours of bead-stimulation (Fig. 5d, i). Thereafter, this fraction continued to increase until reaching saturation (~90%) by 24 h (Fig. 5e, f and 5i), comparable to that seen at pre-patterned AChRs (Fig. 5g, i). In contrast, the overwhelming majority of AChRs outside bead-muscle contacts remained mobile (*D *> 10^-11 ^cm^2^/s; Fig. 5h, i) during this period.

To determine if the bead stimulus that induces AChR clustering locally also exerts long-range effects on receptors outside the contact domain, we examined the diffusion of AChRs in zones at different distance from the beads. Receptors located within < 20 μm or at 20–40 μm and > 40 μm from the beads were tracked and their diffusion coefficients were determined through MSD analyses. Our results showed that receptors at all three distance ranges were equally mobile (Fig. 5j), with nearly identical mean diffusion coefficients. This suggests that the bead-mediated AChR cluster-stimulating signal is confined locally and does not extend beyond the immediate boundary of the contact area.

Collectively, the above results provide direct evidence at single-molecular level for the diffusion-mediated trapping of AChRs during cluster assembly in the muscle membrane. Moreover, they highlight the role of independent, diffusion-mediated movement of single receptors as a driving force for the clustering process.

### AChR movements at developing NMJ

**Figure 6:**
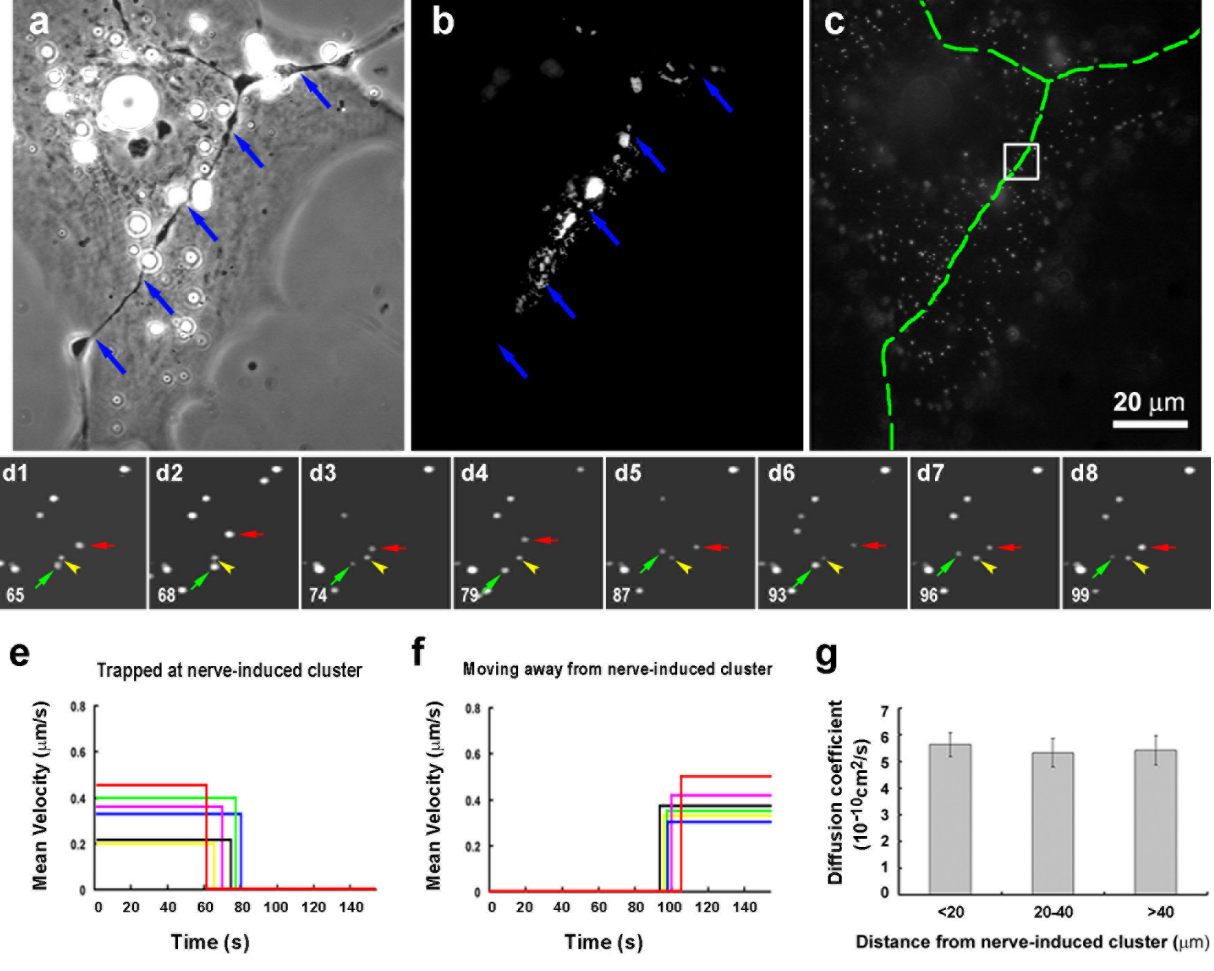
**The effect of innervation on AChR movement**. (a-c) AChR clustering at a nerve-muscle contact (blue arrows in a and b; green dashed line in c) examined with Alexa488-BTX (b) and BBQ (c). (d1–d8) Time-lapse recording of the boxed area in (c). Images were excerpted from a sequence of 130 images. The frame number is indicated at the bottom left corner of the images. Within the AChR cluster some BBQs are immobile (an example indicated by yellow arrowheads) and others are mobile (green arrows). The example of a mobile BBQ that became immobilized is shown by red arrows (trapped at frame d7). (e) The velocity plots of several BBQs at the nerve muscle contact that became immobilized. (f) Examples of trapped BBQs that resumed movement. (g) Mean diffusion coefficients of mobile BBQs at different distances from nerve-muscle contacts: 5.6 ± 0.4, 5.3 ± 0.5, 5.4 ± 0.5 × 10^-10^cm^2^/s at < 20, 20–40, and > 40 μm respectively (n = 31; error bars are standard errors).

In *Xenopus *spinal neuron-muscle cocultures, nerves induce AChR clusters where they touch muscle cells. With Alexa488-BTX-labeling new clusters along nerve-muscle contacts could be readily identified, as shown in Fig. 6, panels a and b, with the corresponding BBQ image shown in Fig. 6c. The synaptic cleft between the nerve and muscle is ~50 nm in width [24], considerably larger than the diameter of QDs used in this study. Within nerve-induced clusters most BBQs were immobile, like those in pre-patterned and bead-induced AChR clusters described above. A small sample area within the cluster domain (Fig. 6c square) is shown in time-lapse recording in Fig. 6d1–d8. In this example, an immobile BBQ can be seen (yellow arrowhead), and also seen are mobile BBQs (Fig. 6d1–d8, green arrows) whose movement was confined to an area more restricted than that of BBQs outside the postsynaptic region. The mobile receptors in postsynaptic clusters typically had *D *on the order of 10^-12 ^to 10^-11 ^cm^2^/s, lower than of those present in the extrasynaptic area, and, additionally, sudden immobilization of these BBQ-labeled receptors was often detected (Fig. 6d1–d8, red arrows; note the cessation of movement from frame d4 onwards). The mean velocity plots of BBQs in Fig 6e show the trapping of several such mobile receptors. Conversely, we also observed the escape of BBQs from clusters, seen as a sudden resumption of their movement (Fig. 6f), which suggests that receptor trapping during NMJ formation is not irreversible.

We also noted that in areas immediately adjacent to postsynaptic AChR clusters, BBQ movement was unrestrained, similar to that of BBQs far from nerve contacts or present on the surface of non-innervated muscle cells, with *D *values ranging from 10^-12 ^to 10^-9 ^cm^2^/s. Calculation of the mean diffusion coefficients of BBQs at different distances from innervation sites (Fig. 6g) clearly demonstrated that the movement of extrasynaptic receptors is independent of their distance from synapses. These results suggest that, like the bead signal, innervation generates a highly localized mechanism for AChR immobilization.

### The effect of F-actin disruption on AChR movement

Previous studies have shown that local F-actin assembly is necessary for the formation of AChR clusters. In fact, dynamic actin polymerization can provide enough motive force for the translocation of entire AChR clusters [10]. Thus, it is of interest to know whether the lateral movement of AChRs at the cell surface is dependent on F-actin assembly. To this end we examined the mobility of single AChRs in cells treated with latrunculin A (LtnA), a marine sponge toxin that blocks actin polymerization by sequestering G-actin. As above, diffusion coefficients were calculated from MSD plots generated from time-lapse BBQ recordings. At a concentration of 40 μM that effectively inhibits AChR clustering [10], LtnA did not significantly alter either the distribution of diffusion coefficients or their mean as compared to the DMSO control (Fig. 3b). This showed that lateral movement of AChRs is diffusion-driven and independent of F-actin cytoskeletal assembly.

### Comparison of AChR and GM1 movement

Because recent studies have suggested that AChRs in the muscle membrane are sequestered into "lipid rafts" enriched in cholesterol and sphingolipids [25,26], we compared the behavior of AChRs and lipid molecules at the cell surface. The B subunit of cholera toxin (CTX) binds specifically to the ganglioside GM1 and can be used as a probe for glycosphingolipid in the plasma membrane [27,28]. Here we applied biotin-conjugated CTX followed by streptavidin-conjugated QDs (Biotin-CTX/QDs, or BCQs) to track GM1 movement.

**Figure 7:**
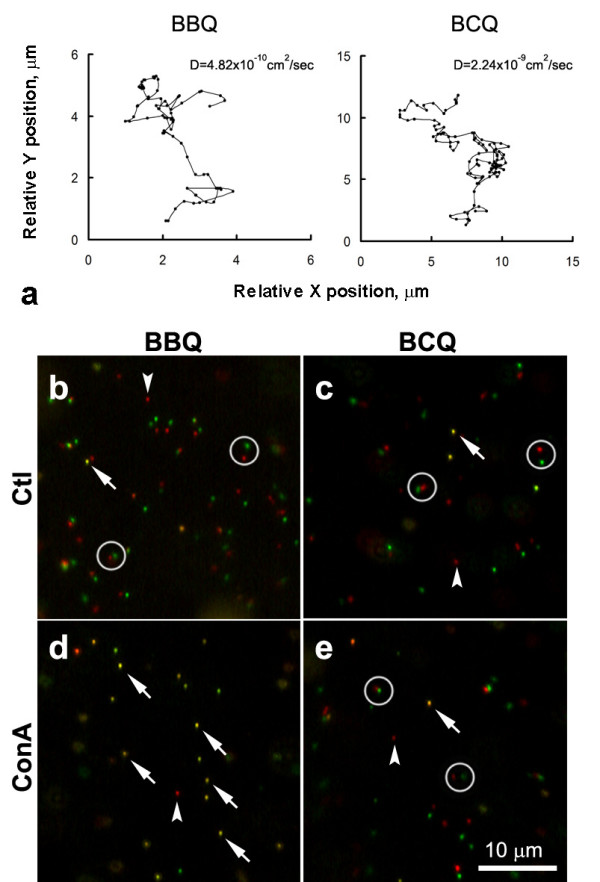
**The comparison between AChR and GM1 movement**. GM1 was labeled with biotinylated cholera toxin plus streptavidin-conjugated QD (BCQ). (a) Trajectories of BBQ and BCQ over an 80-sec recording period. Note the scale of the BCQ plot was 2.5 times that of the BBQ plot. The diffusion coefficient of BCQ was nearly an order of magnitude higher that that of BBQ. (b-e) Mobile and immobile AChRs (b and d) and GM1 (c and e). Two frames in a time-lapse sequence which were separated by an interval of 2 sec were pseudocolored in red and green and superimposed. Mobile QDs appear as paired red and green dots (circles) and immobile ones as single yellow dots (arrows). QDs that blinked during this interval appeared as isolated dots with single color (arrowheads). In control cultures (b and c), diffuse AChRs and GM1 were mobile. ConA treatment resulted in immobilization of AChRs but not GM1 (d and e).

Sample trajectories of a BCQ and a BBQ are shown in Fig. 7a (note the difference in the scaling of the axes). Interestingly, like BBQ (Fig. 7b), BCQ-labeled GM1 lipid molecules also existed in mobile and immobile fractions (Fig. 7c) and the mobile BCQs exhibited diffusion-driven movement in the membrane. The diffusion coefficients of mobile BCQs, however, existed in the narrower range of 10^-10 ^to 10^-9 ^cm^2^/s and were generally an order of magnitude higher than that of BBQs in the wider range of 10^-11 ^to 10^-9 ^cm^2^/s.

To further explore the relationship between AChR and GM1, the effect of Con A on BBQ and BCQ movement was examined. Because this lectin crosslinks AChRs by binding to four mannose residues on the receptors [9], BBQ movement was completely blocked after cells were treated with 100 μg/ml Con A (Fig. 7d). In contrast, BCQ movement was unaffected by this treatment (Fig. 7e), indicating that diffuse AChRs and GM1 move independently of each other.

## Discussion

Using QDs to probe the movement of single AChRs on the surface of muscle cells, we obtained evidence directly supporting the diffusion-trapping model of AChR aggregation at the developing NMJ. By extending the analyses of AChR movement previously carried out with FRAP method with single-particle tracking, this study provided the following new insights on the cluster formation. First, it showed that free diffusion, but not long-range attraction, is the driving force for receptor clustering. Second, the diffusion-driven movement of AChRs is independent of actin polymerization and lipid movement. Third, the cluster grows in size by enlargement of the trapping domain. And fourth, the trap is not a uniformly sticky area but consists of discrete foci at which AChRs are immobilized.

QD labeling of AChRs with the BBQ method is highly specific. It involves high-affinity interactions between streptavidin and biotin and BTX and AChR [29,30]. The specificity is further assured since masking AChRs with unlabeled BTX before BBQ-treatment or using streptavidin-conjugated QDs without pre-labeling with biotinylated-BTX resulted in no QD binding to muscle cells, and the BBQs also did not bind to non-muscle cells which lack muscle AChRs. Extremely bright fluorescence, remarkable photo-stability and nano-size make QDs ideal probes for studying the behavior of single molecules on the cell surface. Despite these obvious advantages, QDs have certain limitations. First, the intrinsic blinking of single QDs interrupts continuous tracking of molecules in time-lapse recording. In addition, a fraction of QDs may exist in non-radiant (dark) form, although the blinking and dark properties are not coupled [31]. Because of the low density of BBQ complexes in our experiments (see Methods and discussed below), it is unlikely that dimers of blinking and dark QDs are associated with the same AChR. In fact, aggregates of QDs associated with crosslinked and internalized AChRs do not blink. Thus, the blinking property is indicative of the singular nature of the molecule being tracked in our study. In addition, single QDs can be distinguished from multiples by the former's relatively uniform fluorescence intensity that is clearly lower than that of the latter. Second, the diameter of QD655 used in this study is 22 nm, which is about 2.5 times the size of a single AChR. While this is a significant size difference, we found that the diffusion coefficient of AChRs estimated from single QD measurements is in the same range as previously estimated using the FRAP technique, which measures the mean behavior of an ensemble of receptors [7,32]. Thus, the attachment of QD had marginal effects if any on receptor movement. This result is also consistent with the notion that the viscosity of the plasma membrane is the main restraint for the movement of QD-bound transmembrane receptors [33]. Lastly, at high BBQ concentration, diffuse AChRs at the cell surface (but not clustered ones) were quickly internalized (data not shown) due to crosslinking as a result of QD-associated streptavidin's binding to multiple biotin-BTX [21]. Aggregates of internalized BBQs could be easily followed within the cytoplasm and their number and size increased with time after the initial labeling, accompanied by a decrease in the BBQ density at the surface. However, this problem was overcome by first labeling AChRs with a mixture of biotin-BTX and Alexa 488-BTX at 1:50 ratio before QD labeling (see Methods). The low density of biotin-BTX-labeled AChRs ensured sparse distribution of QDs on the cell surface to minimize AChR crosslinking via their unoccupied streptavidin sites. Interestingly, in contrast to the Brownian motion exhibited by surface receptors, the internalized receptor aggregates moved linearly along microtubules (Zhang, Geng and Peng, in preparation) and could be unambiguously distinguished from the former.

Single QD tracking allows precise determination of the AChR's diffusion coefficient. Mobile AChRs have *D *on the order of 10^-10 ^cm^2^/s with the fastest reaching 10^-9 ^cm^2^/s. In contrast, AChRs that are essentially immobile have *D *values less than 10^-11 ^cm^2^/s. AChR mobility was found to be age-dependent, with younger cells having significantly faster moving ones (*D *~10^-9 ^cm^2^/s) and older cells having a larger fraction of slow moving and immobile receptors (with *D *less than 10^-11 ^cm^2^/s). While the reason for this change is not known, previous studies have suggested that AChRs interact with the cytoskeleton in a phosphorylation-dependent manner [34]. Thus, it will be of interest to examine whether diffuse AChRs undergo an age-dependent increase in phosphorylation.

To examine the role of diffusion in AChR aggregation, the behavior of single AChRs was examined as they developed into pre-patterned, bead-induced and nerve-induced clusters. By observing the pre-patterned clustering of AChRs with simultaneous fluorescent BTX labeling and QD tracking, we found that this process involves a gradual enlargement of an area of AChR trapping. The final cluster size does not appear to be initially "staked out" but rather develops over a period of several hours, suggesting that a cluster enlarges by encroaching on the neighboring areas to convert them into zones of AChR immobilization. Next, using beads to precisely control the timing and location of AChR cluster induction, we were able to draw several conclusions about the clustering process. First, the bead-muscle contact defines an area of AChR immobilization. Second, clustering does not involve the active attraction of moving AChRs, as evidenced by the random movement of diffuse AChRs and a lack of any bias in their movement toward the beads. Third, QD-labeled AChRs do not slow down even after they enter the clustering site until they suddenly stop at certain points. This suggests that traps induced by beads are made up of discrete foci that immobilize individual AChRs and that only those receptors that come into contact with these points become anchored. Accordingly, AChR density at a developing cluster depends on the number of anchoring points present at the bead-muscle contact at that time. With the low-density BBQ labeling used here to avoid receptor crosslinking, it is likely that only a small subset of anchors was occupied by the BBQ-linked receptors. Future studies with monovalent streptavidin [35] may allow a higher density of AChRs to be tagged by BBQs and should lead to a more dramatic visualization of the trapping of receptors in response to synaptogenic stimulation. Taken together, these findings are in accord with the diffusion-trap hypothesis of AChR clustering.

In this study we also examined AChR clustering at developing NMJs. This proved to be challenging because the timing and exact position of receptor clustering during innervation is not predictable and individual nerve-generated AChR clusters are small compared to pre-patterned and bead-induced clusters. However, by fluorescent BTX and BBQ co-labeling, we were able to examine the mobility of AChRs at and away from nerve-induced clusters. These data, consistent with those obtained with beads, demonstrated the trapping of receptors at nerve-induced clusters and additionally indicated that receptor diffusion within the membrane drives the clustering process. Because diffuse AChRs are present on the muscle surface at high density before innervation, the random Brownian motion of individual receptors will ensure rapid occupancy of anchor points once they appear at nerve-muscle contacts. Thus, our results suggest that the emergence of these anchor points is the rate-limiting step in AChR cluster formation.

What might be the molecular mechanism involved in AChR trapping? First, AChR clustering requires the cytoplasmic protein rapsyn and the assembly of a postsynaptic F-actin cytoskeleton mediated by Rho family small GTPases [11,36]. Rapsyn is a 43 kD cytoplasmic protein that colocalizes with AChRs and associates with them in a 1:1 ratio at the NMJ [2] and in rapsyn's absence no AChR clusters form in muscles [36]. Rapsyn is thought to link AChRs to the cytoskeleton [2] and the clustering of AChRs induced by beads or agrin is impaired when actin polymerization is blocked [10]. Intriguingly, here LtnA-treatment, which potently inhibits actin polymerization, did not affect the mobility of diffuse receptors. This supports the notion that postsynaptic cytoskeletal assembly is mainly for trapping and immobilizing AChRs, possibly via interaction between the AChR-rapsyn complex and the F-actin cytoskeleton, but how the formation of a cytoskeletal scaffold actually produces the foci for receptor anchoring remains to be elucidated. Second, the anchoring foci may depend on the transmembrane kinase MuSK. The earliest step in agrin-induced AChR clustering is MuSK activation and previous studies have shown that MuSK is present at synaptic sites even in rapsyn knockout mice [36,37] where aggregation of AChRs does not occur. It has therefore been suggested that MuSK is part of a primary synaptic scaffold to which rapsyn recruits other components, including AChRs [37]. Third, AChRs could theoretically be trapped by a lipid-based mechanism. Recent studies have suggested that lipid rafts are involved in AChR clustering in rat muscle and in C2C12 myotube cultures [25,26]. However, our results showed that ConA treatment immobilized AChRs but not the glycosphingolipid GM1, a component of the lipid rafts, which suggests that the movement of AChRs in the muscle membrane is not coupled with that of lipid rafts. We also found that in cultured *Xenopus *muscle cells, GM1 was not concentrated at AChR clusters, and that treatment of cells with cholesterol-depleting agents (such as methyl-β-cyclodextrin) did not affect the formation of AChR clusters (Geng and Peng, unpublished data). Thus, the extent to which lipid rafts are involved in generating this postsynaptic specialization at the NMJ warrants further study.

## Conclusion

Single-molecular tracking using QDs has provided direct evidence that the clustering of AChRs in muscle cells in response to synaptogenic stimuli is achieved by two distinct cellular processes: the Brownian motion of receptors in the membrane and their trapping and immobilization at the synaptic specialization. The first is a thermal energy-driven lateral diffusion process that probably requires little cellular regulation and is independent of metabolic energy. The second step is tightly controlled by an elaborate signaling pathway involving the activation of MuSK by nerve secreted factors such as agrin and dependent upon the ensuing cascade of molecular interactions that leads to the assembly of a postsynaptic cytoskeletal scaffold that traps receptors. By coupling these two disparate processes, one passive and the other active, the skeletal muscle cell manages to assemble one of the most spectacular membrane specializations consisting of nearly crystalline two-dimensional packing of AChRs for unfailing synaptic transmission at the NMJ.

## Authors' contributions

LG conducted the experiments involving cell cultures, quantum-dot labeling, microscopy and image processing. LG, HLZ and HBP analyzed the data and put together the illustrations. HBP conceived the study and participated in its design and coordination. All authors read and approved the final manuscript.
